# Ice recrystallisation inhibiting polymers prevent irreversible protein aggregation during solvent-free cryopreservation as additives and as covalent polymer-protein conjugates

**DOI:** 10.1016/j.eurpolymj.2020.110036

**Published:** 2020-11-05

**Authors:** Alice E.R. Fayter, Muhammad Hasan, Thomas R. Congdon, Ioanna Kontopoulou, Matthew I. Gibson

**Affiliations:** a Department of Chemistry, University of Warwick, Coventry CV4 7AL, UK; b Warwick Medical School, University of Warwick, Coventry CV4 7AL, UK

**Keywords:** Polymers, Polymer-protein conjugate, Ice recrystallisation inhibition, Biologics

## Abstract

Protein storage and transport is essential to deliver therapies (biologics), enzymes for biotechnological applications, and underpins fundamental structural and molecular biology. To enable proteins to be stored and transported it is often essential to freeze them, requiring cryoprotectants such as glycerol or trehalose. Here we explore the mechanisms by which poly(vinyl alcohol), PVA, a potent ice recrystallisation inhibitor protects proteins during freeze/thaw to enable solvent-free cryopreservation with a focus on comparing mixing, verses polymer-protein conjugation. A panel of poly(vinyl alcohol)s are investigated including commercial, well-defined (from RAFT), and PVA-protein conjugates, to map out PVA’s efficacy. Enzymatic activity recovery of lactate dehydrogenase was found to correlate with post-thaw aggregation state (less aggregated protein had greater activity), which was modulated by PVA’s ice recrystallisation inhibition activity. This macromolecular cryoprotectant matched the performance of glycerol, but at lower additive concentrations (as low as 1 mg.mL^−1^). It was also demonstrated that storage at −20 °C, rather than −80 °C was possible using PVA as a cryoprotectant, which is not possible with glycerol storage. A second protein, green-fluorescent protein (GFP), was used to enable screening of molecular weight effects and to obtain PVA-GFP bioconjugates. It was observed that covalent attachment of RAFT-derived PVA showed superior cryoprotectant activity compared to simple mixing of the polymer and protein. These results show that PVA is a real alternative to solvent-based protein storage with potential in biotechnology, food and therapeutics. PVA is already approved for many biomedical applications, is low cost and available on a large scale, making it an ideal cryoprotectant formulation enhancer.

## Highlights

• Poly(vinyl alcohol) allows solvent-free protein cryopreservation.
• Lactate dehydrogenase and green fluorescent protein were both successful cryopreserved.
• Poly(vinyl alcohol)-protein conjugates are synthesised and shown to be cryo-stable.

## Graphical abstract

**Figure:**
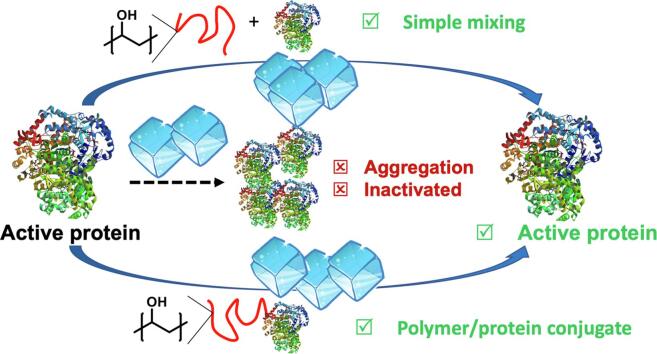

## 1 Introduction

Proteins are essential in fields ranging from biocatalysts for drug development/discovery [1], to emerging therapies (e.g. therapeutic antibodies [2], [3], and in food technology [4]. The study of protein function underpins structural [5], [6] and chemical biology. For example, the biologics market (which includes cell and protein therapies) is estimated at $250B/year and is transforming areas such as oncology [7], [8]. In all cases the protein of interest must be stabilised to ensure it reaches the patient (or point of use) intact and functional, for both therapeutic and economic reasons. Environmental factors such as heat, sunlight and chemical stressors can all lead to denaturation and hence loss of function, meaning freezing is essential to enable storage and remove the need for continuous production. The protein cold chain typically involves either lyophilization or freezing, both of which cause significant stress on the proteins during the cooling cycle by related but distinct mechanisms [9]. For example, there are pH shifts due to selective crystallisation of buffer salts [10], [11], [12], [13], [14], and an increased ice-water interface [15], [16]. Antibodies often aggregate during freeze/thaw cycles and have dramatically altered physicochemical properties [17] and their actual stability varies between each antibody [18] meaning bespoke formulations are required, adding to the final cost. In vaccines the adjuvants are known to lower their freeze-thaw stability preventing long term storage [19] and necessitating a complex supply chain. For example the human papilloma virus (HPV) vaccine [20] contains aluminium salt adjuvants.

Proteins can degrade by three core mechanisms during storage and transport. **i)** Chemical damage, such as oxidation or reaction with excipients; **ii)** Unfolding (denaturing); **iii)** Irreversible aggregation. (i) Is effectively managed by careful storage conditions, but factors (ii) and (iii) need addressing. Current solutions for protein stabilization are the addition of osmolytes such as trehalose [21], [22] or polymer formulations [23] which can replace surface water molecules and make unfolding thermodynamically unfavorable. In research laboratories glycerol is widely used as a cryoprotectant for proteins, as it is simple to add and mix (pipetted as it is a liquid) but it does not give full recovery of function, is viscous and interferes with down-stream assays [24], [25], [26], [27].

To address the process of irreversible protein aggregation during storage, Tibbitt and coworkers have encapsulated enzymes into a photo-reversible hydrogel format. This prevents protein-protein contact, and hence aggregation, and it was shown that they retain their function at ambient temperatures, by preventing this mode of denaturation whilst retaining hydration [28]. Kaplan and coworkers have engineered silk proteins which can retain the activity of enzymes which were dried together as films, and stored for up to 10 months at 37 °C [29]. Similar technology also enabled whole blood storage for diagnostic samples [30]. Maynard has developed trehalose side-chain polymers for protein protection during lyophilization. A range of trehalose-polymers (poly(4,6-*O*-(4-vinylbenzylidene)-α,α-trehalose), polymers of α,α-trehalose modified with a styrenyl acetal, methacrylate acetal, styrenyl ether, or a methacrylate, and poly(5,6‐benzo‐2‐methylene‐1,3‐dioxepane (BMDO)‐*co *‐butyl methacrylate‐trehalose)) have been synthesized and shown to be very potent stabilizers when applied as polymer-protein conjugates [31], [32], [33]. However, the conjugation process itself does result in loss of activity relative to the free protein alone, so the benefit of free polymer at higher concentrations, verses conjugated at lower must be considered, and any new polymer requires safety and approval processes for biomedical applications.

During the freezing of proteins, ice crystals form and exclude all other solutes (as ice forms a pure phase) meaning the concentration of all the solutes effectively increases, known as cryo-concentration [34]. Furthermore, as the ice crystals grow (recrystallisation) the effective surface area (i.e. interfaces where proteins are located) decreases, meaning that protein-protein contacts increase and hence aggregation is more likely. In Nature ice recrystallisation is prevented by the production of antifreeze proteins [35] which are potent ice recrystallisation inhibitors (IRIs) and can function at sub 1 mg.mL^−1^ concentrations [36]. However, antifreeze proteins are not ideal for many biotechnological applications, not least due to their cost compared to current technologies such as glycerol. Therefore there is significant interest in developing polymer mimics of antifreeze proteins, which has been extensively reviewed [37], [38], [39] as well as small molecule inhibitors [40]. The most potent IRI polymer is poly(vinyl alcohol) which is synthetically scalable, low cost, has a well-established toxicity profile and is approved for several formulation and food based applications [41]. Due to its high IRI activity PVA has been widely explored [42], [43], [44], [45], including for cell storage applications [25], [46]. It should be noted that PVA’s exceptional activity is still the subject of extensive research [47], and that IRI is a very rare property in polymers. Gibson and co-workers have shown that PVA can be used for the solvent-free cryopreservation of enzymes when used in combination with poly(ethylene glycol) and can match the performance of trehalose or glycerol based methods [48]. The exact mechanism and limitations of the use of polymers as ice-controlling excipients as a next-generation tool for the storage of proteins has not yet been fully explored.

Considering the above, this work explores the use of PVA for cryopreserving proteins both as an additive and as a covalent polymer/protein conjugate. Firstly, the enzyme LDH, which is challenging to cryopreserve by conventional methods, is shown to be protected by PVA across a range of conditions. The mechanism of action is linked to preventing the formation of protein aggregates. Secondly, a higher-throughput assay is established using green fluorescent protein, and a panel of well-defined PVA’s (from RAFT) were screened for activity. Finally, PVA-GFP conjugates were synthesized and shown to lead to protection even across many freeze/thaw cycles. This conclusively shows that IRI active polymers have significant potential for protein-based storage applications.

## 2 Results and discussion

**Fig. 1:**
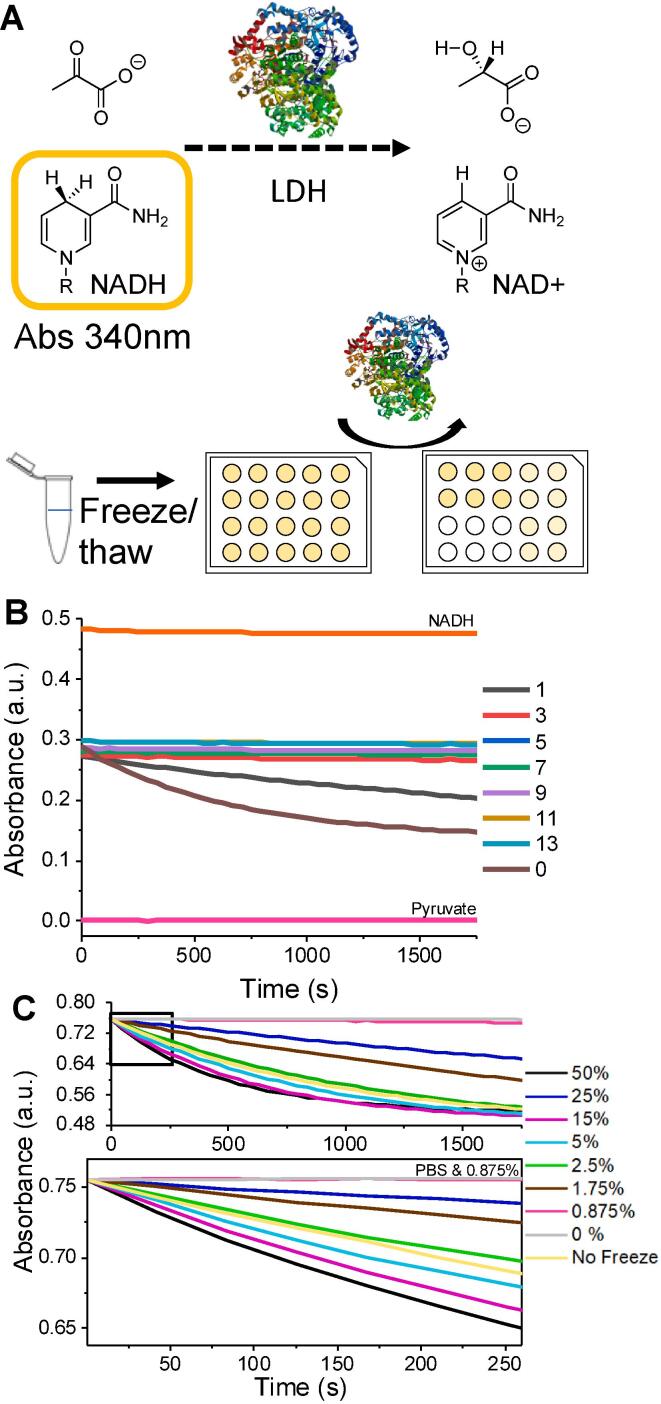
LDH cryopreservation screening. A) Assay to determine LDH activity. Reduction of NADH to NAD+ with corresponding decrease in absorbance at 340 nm; B) Activity retention (or loss) of LDH after the indicated number of freeze/thaw cycles; C) Impact of glycerol concentration on recovery after a single freeze/thaw cycle. [Glycerol] reported as % v/v. Freeze/thaw cycles were Liq. N_2_/37 °C.

Lactate dehydrogenase (LDH) is a challenging enzyme to cryopreserve (and biotechnologically important) [49], [50] and was hence chosen as the protein of interest here to explore the use of PVA’s ice recrystallisation inhibition (IRI) activity for solvent-free protein storage. Fig. 1A shows the assay for measuring LDH activity post-thaw; the conversion of pyruvate to lactate, with corresponding oxidation of nicotinamide adenine dinucleotide (NADH) to NAD+, which is measured by monitoring the decrease in absorbance at 340 nm. This reaction is rapid and hence low (0.031 nM) concentration of LDH in the final assay are required to enable comparison of the rate of reaction. Control experiments where LHD was exposed to up to 13 freeze/thaw cycles are shown in Fig. 1B. Freeze/thaw with the addition of a range of glycerol concentrations are shown in Fig. 1B and 1C to establish a control. As would be expected, addition of no cryoprotectant leads to loss of activity upon thawing and there is a non-linear relationship between glycerol concentration and cryoprotectant outcome. The initial slope of the curve is used for comparison from this point on.

**Fig. 2:**
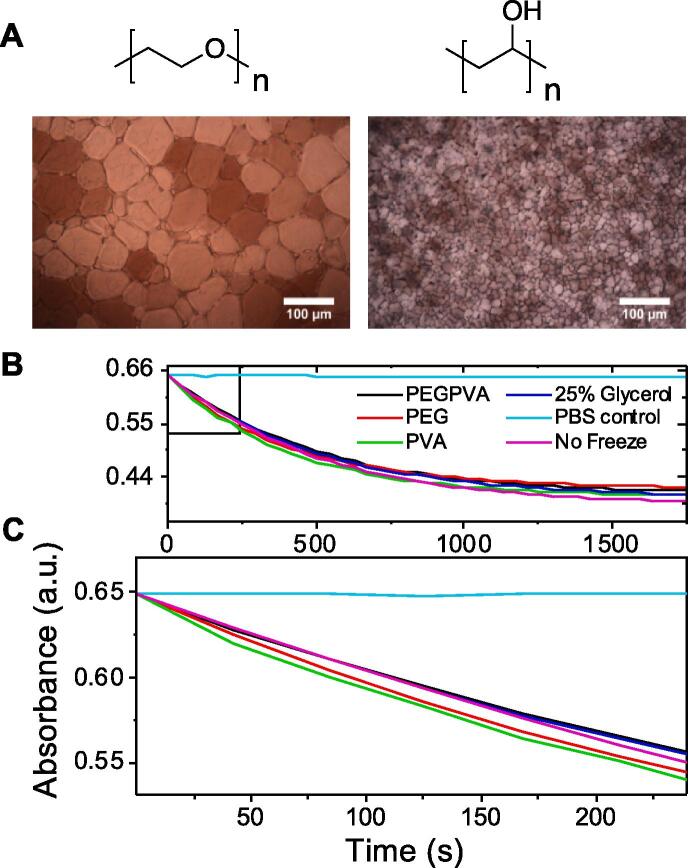
A) Chemical structure of PEG/PVA and representative ice wafers grown with each polymer [PEG] = 10 mg.mL^−1^, [PVA] = 1 mg.mL^−1^; B) LDH activity recovery in the (PEG = 100 mg.mL^−1^, PVA = 1 mg.mL^−1^) after 5 FT cycles (−196 to 25 °C), recorded by catalysis of NADH to NAD+, observed by a decrease in absorbance, compared to a no CPA control (buffer); C) Zoom in of linear region.

The primary aim here was to explore how IRI active polymers modulate protein recovery by controlling ice crystal growth. Fig. 2A shows example ice wafers which have been grown with added PVA or PEG (negative control with no IRI^36^) to highlight the unique IRI activity of PVA. In brief, small ice crystals after annealing at sub-zero temperatures indicates more IRI activity. Full dose-dependent profiles have been previously reported and are not repeated here [36], [44]. Typically PVA inhibits all growth below 1 mg.mL^−1^ unless very low (<500 g.mol^−1^) molecular weights are used [44], [51].

The impact of IRI active/inactive polymers on freeze/thaw recovery of LDH was evaluated, Fig. 2B/C. 100 mg.mL^−1^ PEG (~10 wt%, red) showed some cryoprotective benefit. At this high concentration most additives would have an impact and this concentration of PEG has been previously reported for LDH cryopreservation [49]. PVA, in contrast, at just 1 mg.mL^−1^ (0.1 wt%, green) gave the same recovery as 25 wt% glycerol or 100 mg.mL^−1^ PEG, highlighting its potency. Increasing to seven freeze/thaw cycles showed that glycerol performed worse than all the polymer formulations under these conditions Figure S1). One possible explanation for glycerol’s poorer performance under repeated cycles is that the protein is in contact with the solution of cryoprotectant at raised temperatures, which may lead to denaturation, even if it is also protecting against cold stress. Previous reports of using IRI active polymers for protein storage [48] found that for some proteins an additional hydrophilic polymer was required to achieve protection, but the observations made here suggest the exact formulations needed for practical storage may vary between proteins and that a minimal solution of just PVA may be sufficient for some, which is desirable.

**Fig. 3:**
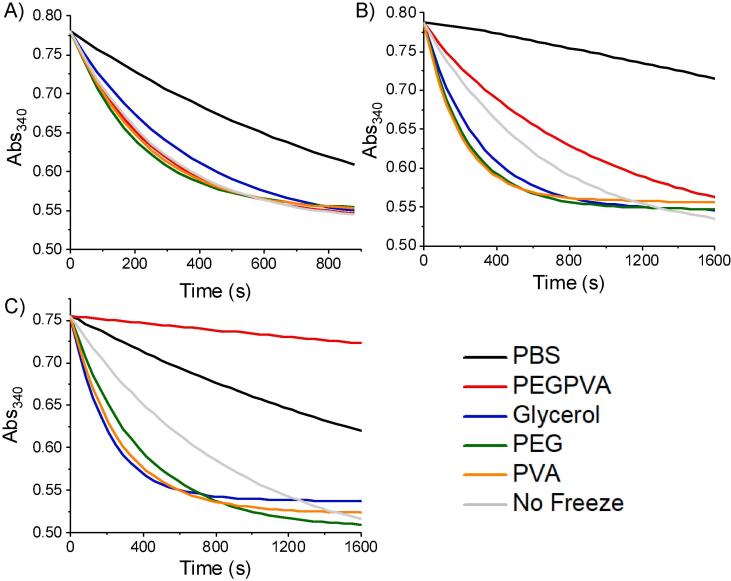
Variable storage period analysis. All samples were plunge frozen into Liq. N_2_, stored at −20 °C for indicated period of time and then thawed at 37 °C. A) 24 h; B) 7 days; C) 1 month. PEG = 100 mg.mL^−1^, PVA = 1 mg.mL^−1.^

The above data shows that IRI active polymers can be employed to prevent freeze/thaw induced deactivation of LDH. However, it is important to comment that the experiments were conducted under conditions that are impractical and not truly representative of routine use in a laboratory. Therefore, we set out to look at the impact of storing the proteins at −20 °C (i.e. the temperature of a standard laboratory freezer) for 1 day to 1 month, as a truly rigorous test of the utility of this technology, and to show the potential for reducing the need for energy intensive −80 °C freezers, Fig. 3. The samples were prepared as above and plunged into Liq. N_2_ then transferred to the −20 °C freezer. Note, this was crucial to ensure the samples freeze, and to avoid unfair comparisons (glycerol solutions would take a long time to freeze directly in −20 °C and we did not want to bias cryopreservation results due to protein denaturation due to extended contact with liquid glycerol). After 24 h storage and thawing, all samples performed well with some retention of activity – notably, even the PBS control gave some protection (thus highlighting that proteins themselves can survive some cold stress intrinsically). After 7 days storage at − 20 °C glycerol continued to perform well but the PEG/PVA mixtures began to lose activity, which upon 28 days storage performed poorly. All other formulations retained some activity at 28 days, and interestingly the PVA at just 1 mg.mL^−1^ (~0.1 wt%) successfully protected the LDH, giving the same results as PEG at 100 × higher concentration. This analysis shows that PVA is indeed a potent protein cryoprotectant, which functions at concentrations far lower than other polymers or indeed the current standard of glycerol, but that some additional formulation may be required for an individual protein sample, to achieve optimum results.

**Fig. 4:**
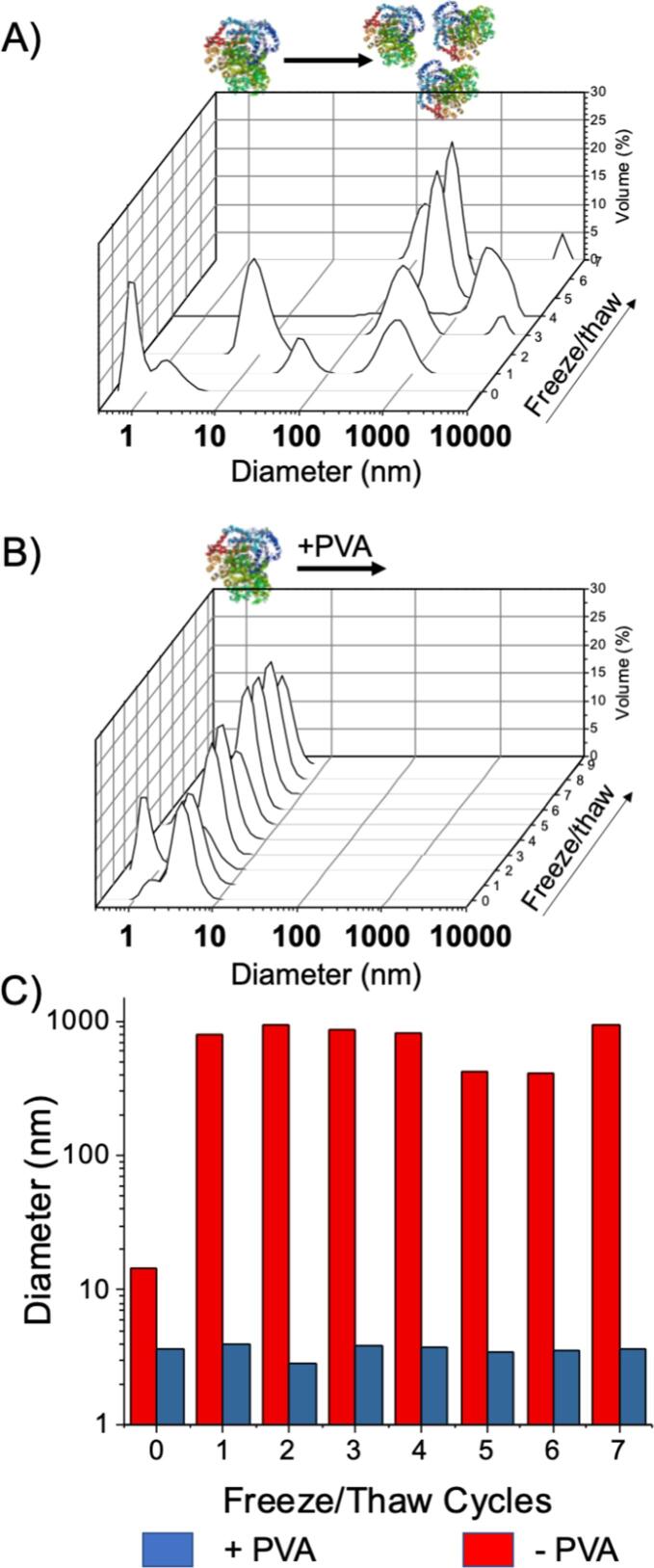
Dynamic light scattering analysis of freeze/thaw induced aggregation of LDH and impact of PVA. A) LDH size distribution over 7 freeze/thaw cycles with (right) and without (left) PVA; B) LDH size distribution over 7 freeze/thaw cycles. [PVA] = 1 mg.mL^−1^ (Freeze/Thaw cycle was Liq. N_2_/25 °C).

To validate the hypothesis that irreversible protein aggregation was a primary mechanism for loss of enzyme activity, dynamic light scattering (DLS) was used. If PVA is inhibiting ice growth, and hence reducing the effective protein concentration at ice crystal surfaces, then there will be less protein aggregation (due to fewer protein-protein contacts). Hence as DLS is sensitive to size (hydrodynamic diameter) this effect can be probed. Fig. 4A shows that before freezing only particles (protein) below 10 nm were observed, but after freeze/thaw the diameters dramatically increased to >1000 nm. This correlates well with the LDH enzymatic activity data. In contrast to LDH in buffer, a solution of LDH with just 1 mg.mL^−1^ PVA showed essentially no aggregation over 7 freeze/thaw cycles and confirms that aggregation prevention is the (or at least part of) the mechanism of cryoprotection by IRI active polymers, Fig. 4B. [Note, the exact size/distribution in Fig. 4B shows some variance in diameter and intensity as samples were withdrawn from a master batch to take DLS (rather than the same vial being freeze/thawed), which leads to some variability, but still demonstrates the principle.] The observations made here are significant as many protein-based pharmaceuticals cannot be frozen due to aggregation, including insulin, which can form amyloid – like aggregates [52] and vaccines, which cannot be stored frozen [19]. It should be noted, that there are previous reports showing that slow freezing of proteins (to encourage larger crystals) performed better than fast freezing (to produce smaller crystals). The hypothesis was that reducing protein contact with the ice surface is preferential [50]. This is in contrast to our results, although the same work did note that recrystallisation upon thawing was a problem, which our polymers also mitigate. Hence the benefits seen here could be associated with balancing the different denaturation pathways.

The above data demonstrated that IRI active polymers protect LDH from freeze-induced damage by preventing irreversible aggregation. There does remain, however, the question of if the polymer in solution is required, or if covalent conjugation of the polymer to the protein gives the most protection, by effectively concentrating the polymer at the protein surface? Answering this may also bring understanding into the underpinning mechanism of protection. PVA itself is appealing for this application due to its widespread use in formulations and low cost. To enable us to probe if PVA–protein conjugates are cryo-stable, and to gain more detail about how the absolute magnitude of IRI activity of the polymers can impact the cryopreservation outcomes, an alternative protein was used which also enabled more high-throughput testing. Green fluorescent protein (GFP) was selected as the probe, and was produced by recombinant expression in *E. coli*. GFP Fluoresces (excitation at 395 nm, emission at 509 nm) when correctly folded, and upon denaturation the fluorescence intensity decreases. It is important, however, to note that GFP is itself relatively freeze tolerant, so multiple cycles (below) are used. We have previously reported that PVA used in combination with PEG can protect GFP [48] using commercial PVA. For the polymer-protein conjugation studies conducted here PVA will be the only additive (as the covalent conjugate), so it was first essential to study the role of PVA as the sole additive without conjugation, to establish a baseline.

**Fig. 5:**
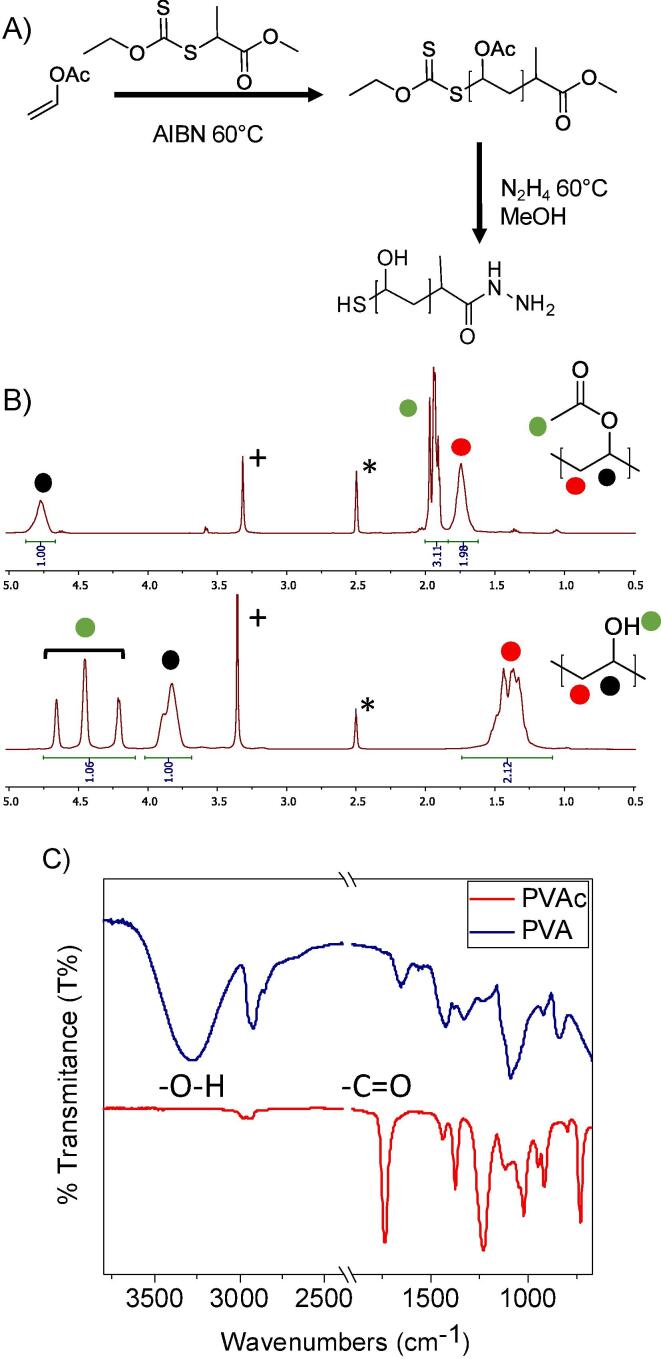
Synthesis and characterization of PVAc/PVA. A) RAFT/MADIX polymerization of vinyl acetate, followed by deacetylation; B) ^1^H NMR analysis showing removal of acetate protecting group * residual DMSO, + residual HOD; C) FTIR of before/after acetate removal.

For this part of the study, a panel of PVA’s were synthesised using RAFT (Reversible Addition-Fragmentation Chain Transfer) polymerization, Table 1. Vinyl acetate was polymerised using 2-(ethoxycarbonothioyl)sulfanyl propanoate (EXEP) in bulk. Conversion was determined by ^1^H NMR, and the higher [M]:[CTA] resulted in lower conversions, as would be expected for lesser activated monomers (LAM) in bulk polymerisation. SEC of the PVAc’s revealed monomodal distributions, but broader than would be expected for normal RAFT, due to the nature of VAc as a LAM. The acetate groups were quantitatively removed by hydrazine to generate PVA, as confirmed by IR and ^1^H NMR spectroscopy, Fig. 5.

**Table 1:** Poly(vinyl alcohol)/poly(vinyl acetate)s synthesised here.

PVAc Code	[M]:[I]	Conversion^(a)^ (%)	Mn_Theo_^(b)^ (g.mol^−1^)	Mn_SEC_^(c)^ (g.mol^−1^)	*Đ_SEC_*^(c)^	PVA Code
PVAc 21	25:1	82	1800	2000	1.34	PVA 21
PVAc 71	100:1	80	6900	6400	1.40	PVA 71
PVAc 100	125:1	71	7600	8800	1.61	PVA 100
PVAc 148	200:1	63	8000	13,000	1.66	PVA 148
PVAc 183	250:1	54	11,600	16,000	1.63	PVA 183
PVAc 294	1000:1	32	27,500	25,600	1.80	PVA 294

(a) From ^1^H NMR; (b) predicted from conversion and [M]:[I] ratio; (c) From size exclusion chromatography in DMF verses PMMA standards.

**Fig. 6:**
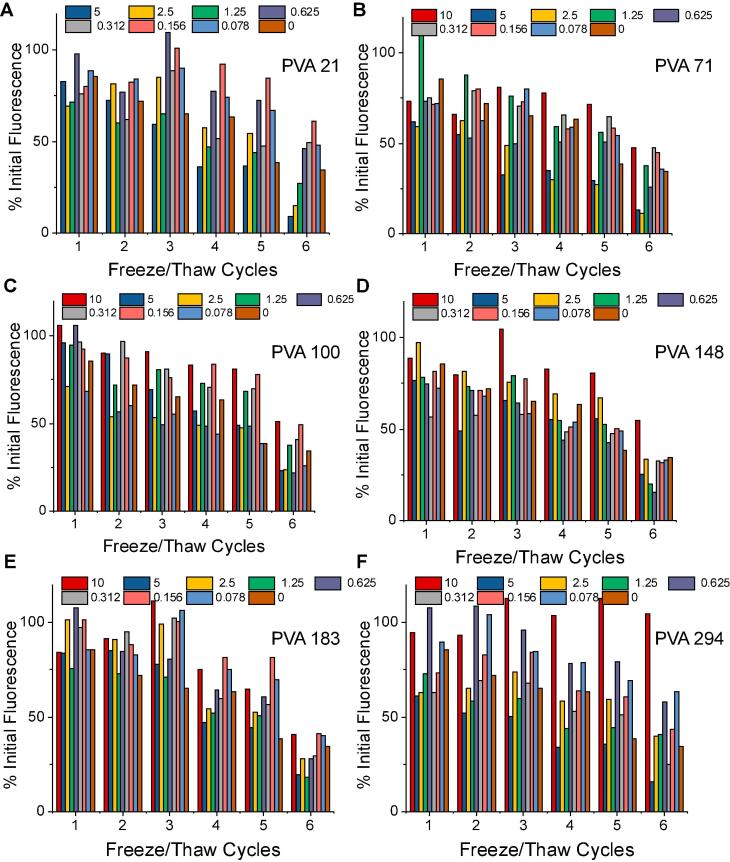
Fluorescence intensity at ex/em 395/509 nm for various PVA polymers at different concentrations, reported at percentages of initial recorded fluorescence; A) PVA21; B) PVA71; C) PVA100; D) PVA148; E) PVA183; F) PVA294.

To evaluate the cryoprotective properties of the PVA, GFP was prepared in PBS at 1 µM and a dilution series of each polymer added. The solutions were frozen to −20 °C, and then allowed to thaw at room temperature. After each freeze/thaw cycle, fluorescence was measured at excitation of 395 nm, emission at 509 nm and reported relative to the starting fluorescence. It is crucial to note that this method enabled testing of the same sample after each cycle, whereas an enzymatic assay would require removal of some sample, or using a very large number of individual replicates, and hence this GFP method enabled screening. The data is reported in Fig. 6. Without any polymer additives, GFP alone loses its fluorescence and after 6 cycles it has fallen to below 50% of its starting value.

The data in Fig. 6 confirmed that PVA can mitigate the damage to GFP upon freeze-stress and that there is a dose-dependency to its function with excess PVA being detrimental. Bell shaped responses to antifreeze protein (or PVA) concentration in cellular cryopreservation has been reported [46], [53], [54] due to needle–like ice crystals damaging cell membranes, but this seems unlikely to be a problem for protein storage. There was no clear molecular weight dependency but it appeared the shorter polymers may have some benefit compared to longer. This could be due to aggregation of the PVA itself, as higher molecular weights are known to form gel particles during freeze/thaw cycling [55], [56]. It is important to note that here PEG was not added as a secondary cryoprotectant, unlike in previous reports of IRI-driven protein stabilisation where the secondary non–IRI active polymer enhanced its function [48]. This experimental condition was essential to enable evaluation of the role of PVA, rather than to obtain the ‘best’ system and to guide parameters for the polymer-protein conjugation (below) where only a single polymer would be added. The data does confirm that PVA has a beneficial effect and that the formulation of the PVA conditions are essential to each protein used, but that in general lower molecular weights are preferred, below 10 kg.mol^−1^ [48].

From the above we cannot rule out any other roles of the polymer in stabilising, e.g. through hydrophobic contacts. Maynard has shown that trehalose-functional polymers can protect various proteins during freeze stress when covalent conjugated to proteins, and their efficacy is reduced when added free in solution [31], [32]. Therefore, a strategy was devised to conjugate PVA to GFP. It should be noted that conjugation of PVA is extremely challenging as it must be obtained from a precursor polymer (PVAc) and the acetate groups removed (under basic conditions). There is only a single previous report of PVA-protein conjugation using commercial disperse PVA [57].

**Fig. 7:**
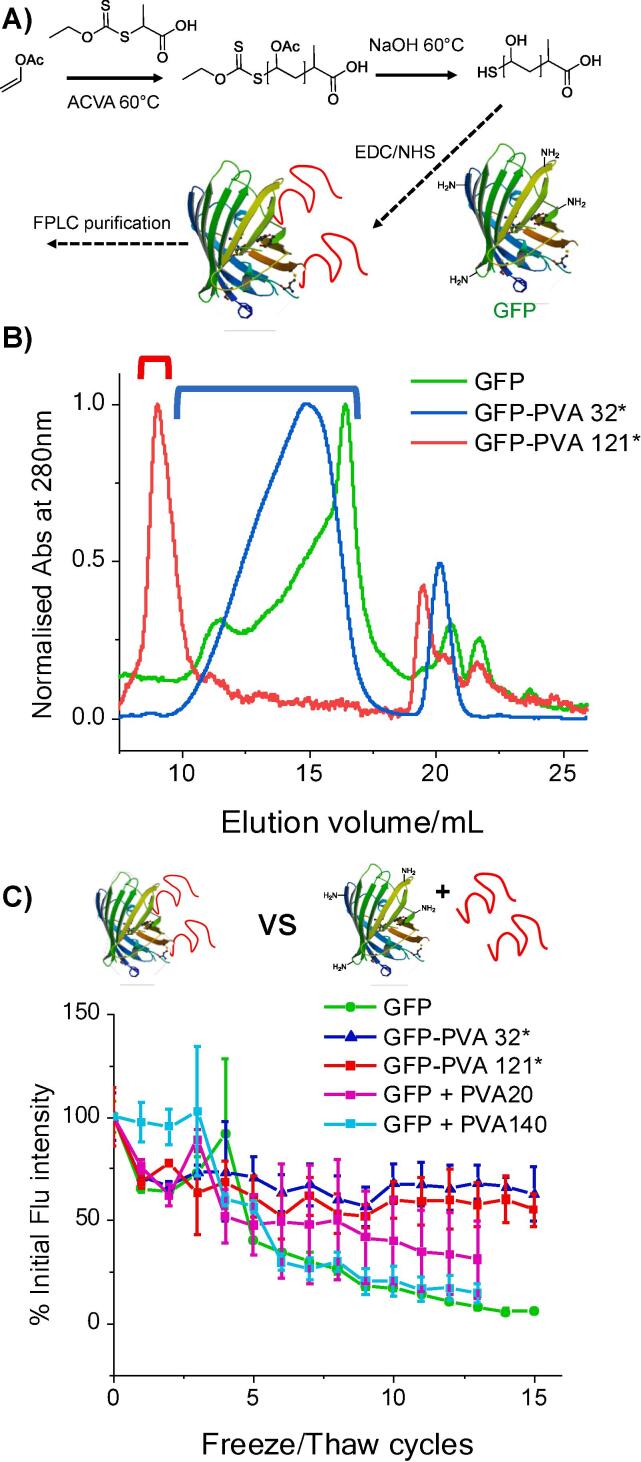
PVA-GFP bioconjugation and freeze/thaw tolerance. A) Synthetic strategy for conjugation of PVA to GFP via EDC/NHS coupling; B) FPLC proof of conjugation and indicated fractions used for purification; C) % Fluorescence recovery of GPF fluorescence after freeze/thaw cycles for GFP, PVA conjugated, and PVA mixed (non-conjugated). [PVA] = 0.2 mg.mL^−1^.

To generate a PVA which could be conjugated via a chain end RAFT/MADIX was employed (as above), but deprotection was achieved using NaOH to generate a carboxylic acid end-group from the terminal carboxylic acid ester (as opposed to hydrazide when hydrazine is used). Two model polymers were prepared as shown in Table 2. These polymers were conjugated to GFP using EDC/NHS coupling for targeting of surface lysine residues by adding a large excess of the polymer, Fig. 7A. After conducting the conjugation reaction in pH 6 phosphate buffer for 12 h, purification was achieved using FPLC (fast protein liquid chromatography), Fig. 7B. GFP in solution always forms some aggregates which can be seen at 20 mL. After conjugation a shift to shorter elution times, consistent with an increase in molecular weight was observed, and the indicated fractions were collected. By using UV/Vis spectroscopy to measure (at 280 nm) the protein concentration in a known weight of purified conjugate, the average number of PVA chains attached to each GFP was determined to be 15 for GFP-PVA32 and 11 for GFP-PVA121. These were then assayed for freeze/thaw protection as described above. Importantly the conjugation process did itself not lead to any reduction in fluorescence compared to (lyophilized) protein alone (Figure S3. Initial loss of fluorescence after the first thaw was observed for the conjugates and the GFP control, and the PVA32 GFP mixture, but not with PVA121 GFP Fig. 7C. Subsequent freeze/thaw cycles showed that the fluorescence activity of the conjugates was essentially unaffected after the initial loss, compared to the PVA GFP mixtures and the GFP control which consistently lost activity with each cycle and after 5 cycles dropped to 40% of the initial fluorescence. After extended testing of 15 cycles, GFP-PVA conjugates retained fluorescence output with no further loss of activity, whilst GFP-alone had essentially lost all almost activity (6% of initial fluorescence) and the PVA-GFP mixtures had dropped to similar levels.

**Table 2:** Poly(vinyl acetate)/Poly(vinyl alcohol) synthesised for bioconjugation.

PVAc Code	[M]:[I]	Conversion (%)	Mn_Theo_ (g.mol^−1^)	Mn_SEC_ (g.mol^−1^)	*Đ*_GPC_	PVA Code
PVAc 32*	30:1	90	2300	2760	1.31	PVA 32*
PVAc121*	150:1	76	9800	10,500	1.48	PVA 121*

This data shows that covalent conjugation of PVA is a potent tool for protein cryoprotection. This beneficial effect might be due to localisation, whereby the polymers are present at high concentration on the protein surface and locally controlling ice growth, or a combination of this and general stabilisation against unfolding and aggregation due to the steric bulk of conjugated polymer. PVA is also appealing due to its low cost and known safety profile unlike bespoke new polymers, and hence this approach may be suitable for application to freeze-sensitive proteins, especially as the conjugation method is straightforward and does not require site-specific modification.

## 3 Conclusions

Herein we report a detailed study on the use of poly(vinyl alcohol), PVA, as a cryoprotectant for protein storage based on its potent ice recrystallisation inhibition (IRI) activity. Its ability to cryopreserve two different proteins (lactate dehydrogenase and green fluorescent protein) as both an additive and as a covalent polymer/protein conjugate is demonstrated. Using lactate dehydrogenase, PVA was shown to enable cryopreservation at just 1 mg.mL^−1^, matching, or out-performing glycerol, which required significantly higher concentrations to protect the protein. Activity was retained even through multiple freeze/thaw cycles, or storage at −20 °C, which is relevant for routine laboratory usage. It is shown that LDH recovery correlated with the inhibition of irreversible aggregation, supporting the hypothesis that ice recrystallisation drives protein aggregation during cryostorage. To further probe this, a high-throughput assay using green-fluorescent protein as the read-out was employed to screen the impact of PVA molecular weight on cryopreservation. Using a panel of PVA’s derived from RAFT/MADIX it was seen that all PVAs can provide some protection but that the strong molecular weight dependent trends observed with the IRI activity of PVA were not present here, and that all molecular weights performed similarly, with PVA concentration being main factor. Finally, a strategy was devised to enable non-specific PVA conjugation to GFP using RAFT polymerization. The polymer-protein conjugate was more stable to freeze/thaw stress than protein with the same amount of non-conjugated PVA, with no appreciable loss or change in protein activity even after fifteen freeze thaw cycles. These results show that the structurally simple, low cost and widely – used polymer PVA is indeed a potent macromolecular cryoprotectant for proteins. This may help design innovative storage formulations across a range of application areas from vaccines to biocatalysts.

## 4 Experimental section

### 4.1 Materials

All chemicals were used as supplied. Ethyl acetate, hexane, methanol, Petroleum ether 40–60 °C dichloromethane and magnesium sulphate were all purchased from Fisher Scientific at laboratory reagent grade. Deuterated chloroform (99.8 atom %D), dimethyl sulfoxide-d6 (99.9 atom %D), vinyl acetate (97.0%), 4,4′-azo-bis(4-cyanovaleric acid) (≥80.0%), 2,2′-azo-bis(2-methylpropionitrile) (98%), potassium ethyl xanthate (96%), 2-(methyl bromopropionate) (98%), aqueous hydrazine hydrate solution (50–60%), PBS buffer (preformulated tablets, yielding 0.01 M phosphate buffer, 0.0027 M potassium chloride and 0.137 M sodium chloride, pH 7.4), *N*-Ethyl-*N*′-(3-dimethylaminopropyl)carbodiimide hydrochloride (EDC.HCl, >99.0%), *N*-hydroxy succinimide (NHS, 98%), imidazole (>99%), β-nicotinamide adenine dinucleotide, reduced disodium salt hydrate (NADH 97%), sodium pyruvate (ReagentPlus > 99%), polyethylene glycol (BioUltra 4000), poly(vinyl alcohol) (MW 9–10 kDa, 80% hydrolysed) and SealPlate films were purchased from Sigma Aldrich. L–lactate dehydrogenase was purchased from Roche. MilliQ water (18.2 mΩ).

Protein expression and purification are described in the Supporting Information.

### 4.2 Physical and analytical methods

Ice recrystallisation inhibition (‘splat’) assay is described in the supporting information.

#### 4.2.1 NMR spectroscopy

^1^H and ^13^C NMR spectra were recorded at 400 MHz on a Bruker DPX – 400 spectrometer respectively, using deuterated solvents purchased from Sigma Aldrich. Chemical shifts of protons are reported as δ in parts per million (ppm) and are relative to tetramethylsilane (TMS) at *δ* = 0 ppm when using DMSO or solvent residual peak (CH3OH, *δ* = 3.31 ppm/ DMSO, *δ* = 2.50 ppm/ D2O, *δ* = 4.79 ppm).

#### 4.2.2 Size exclusion chromatography

Size exclusion chromatography (SEC) analysis was performed on an Agilent Infinity II MDS instrument equipped with differential refractive index (DRI), viscometry (VS), dual angle light scatter (LS) and variable wavelength UV detectors. The system was equipped with 2x PLgel Mixed C columns (300 × 7.5 mm) and a PLgel 5 µm guard column. The eluent is CHCl3 with 2% TEA (triethylamine). Samples were run at 1 mL/min at 30 °C. Poly(methyl methacrylate), and polystyrene standards (Agilent EasyVials) were used for calibration. Analyte samples were filtered through a GVHP membrane with 0.22 μm pore size before injection. Respectively, experimental molar mass (Mn, SEC) and dispersity (Đ) values of synthesized polymers were determined by conventional calibration using Agilent GPC/SEC software.

### 4.3 Protein assays

#### 4.3.1 LDH freeze/thaw assay

NADH and sodium pyruvate were made up to stock concentrations of 63 mM and 10 mM respectively. NADH (4 µL) and sodium pyruvate (10 µL) were added to 1 mL PBS to make the reaction buffer. All potential cryoprotectants (glycerol, 4 kDa PEG, 10 kDa PVA, PEG/PVA) were added to LDH in a 50:50 vol and frozen in triplicate at the chosen temperature (−196 °C or −20 °C). The samples were thawed at 25 °C after ≥1 cycles. 5 µL of CPA:LDH samples were added to a 96 well plate and diluted by the addition of the reaction buffer (195 µL) to give a final protein concentration of 0.031 nM. This concentration was essential to allow the rate of reaction to be observed due to the rapid kinetics of this enzyme. Absorbance at 340 nm was recorded over 30 min at 25 °C using a BioTek Synergy HTX multimode reader and compared to that of an unfrozen LDH control and unreacted NADH.

#### 4.3.2 GFP freeze/thaw assay

PVA and GFP was made up to a stock concentrations of 11 mg.mL^−1^ and 30 µg.mL^−1^ respectively in PBS buffer (0.1 M, pH 7.4). PVA stock solution was added in serial dilution (180 µL) to a black 96 well plate in triplicate. GFP (20 µL) was then added to each well and the plate sealed with SealPlate film. Fluorescence was recorded at 25 °C using a BioTek Synergy HTX multimode reader using an excitation wavelength of 395 nm and an emission wavelength of 509 nm. Samples were then frozen at −20 °C and then thawed at ambient temperature. The fluorescence recorded as above as compared to that of the unfrozen GFP PVA solutions.

### 4.4 Synthetic methods

PVA and PVAc would synthesized according to previously reported protocols [44], and are described fully in the Supporting Information.

#### 4.4.1 Synthesis of PVA-GFP conjugates

As a representative example, PVA32 (0.2 g, 1400 g.mol^−1^, 400 eq protein, (25 eq surface lysine)), EDC. HCl (10 mg, 8 × 10^−5^ mol, 400 eq protein) and *N*-hydroxysuccinimide (8 mg, 8 × 10^−5^ mol, 400 eq protein) were dissolved in phosphate buffer (pH6, 5 mL) at 4 °C and allowed to stir for 5 min. GFP (10 mg, 0.38 mL of 1 mM stock solution, 1 eq protein, (17 eq surface lysine)) was added and the reaction allowed to proceed at 4 °C for 8 h. The reaction was then concentrated by centrifugal dialysis (MWCO 10 kDa) (note: 30 kDa was used for larger PVA conjugates). Concentrated solutions were then purified using FPLC in PBS buffer (0.1 M, pH 7.4) by collecting the fractions relating to PVA-GFP conjugate. Conjugates were characterised by a combination of FPLC and UV/Vis spectroscopy of the dialysed and freeze-dried sample, to calculate the protein concentration in the overall mass of the conjugate to determine degree of conjugation.

## CRediT authorship contribution statement

**Alice E.R. Fayter:** Investigation, Methodology, Writing - original draft. **Muhammad Hasan:** Investigation. **Thomas R. Congdon:** Investigation, Methodology. **Ioanna Kontopoulou:** Investigation. **Matthew I. Gibson:** Conceptualization, Supervision, Writing - review & editing.

## Declaration of Competing Interest

The authors declare the following financial interests/personal relationships which may be considered as potential competing interests: MIG, AERF and MH are named inventors on a patent application relating to this work.
