# Supplementary material for: Ice recrystallisation inhibiting polymers prevent irreversible protein aggregation during solvent-free cryopreservation as additives and as covalent polymer-protein conjugates
Source: Eur Polym J. 2020 Nov 5;140:110036. doi: 10.1016/j.eurpolymj.2020.110036 (PMC7709485; doi:10.1016/j.eurpolymj.2020.110036)
Supplement: Supplementary data 1 [file mmc1.pdf]

Supporting Information for

Ice Recrystallisation Inhibiting Polymers Prevent Irreversible Protein Aggregation During Solvent-Free Cryopreservation as the Primary Mechanism of Action; Additives, and as Polymer-Protein Conjugates

Alice E.R. Fayter,[a] Muhammad Hasan,[a] Thomas Congdon,[a] Ioanna Kontopoulou[a] and Matthew I. Gibson [a,b]

a) Department of Chemistry, University of Warwick, Coventry, CV4 7AL, UK,

b) Warwick Medical School, University of Warwick, Coventry, CV4 7AL, UK

Corresponding Author Email, m.i.gibson@warwick.ac.uk

Additional Experimental Details

FT-IR Spectroscopy.

The Fourier transform-infrared (FT-IR) analysis was performed on Agilent Cary 630 FTIR spectrometer with diamond ATR system, in the range of 650 to 4000 cm^{-1}.

Fast protein liquid chromatography.

Fast protein liquid chromatography (FPLC) was performed using AKTA pure (GE Healthcare) with a flow rate of 1 $mL.min^{-1}$ using PBS buffer (0.1M, pH 7.4) as the eluent.

Splat Ice Recrystallisation Inhibition Assay.

Ice wafers were annealed on a Linkam Biological Cryostage BCS196 with T95-Linkpad system controller equipped with a LNP95-Liquid nitrogen cooling pump, using liquid nitrogen as the coolant (Linkam Scientific Instruments UK, Surrey, U.K.). An Olympus CX41 microscope equipped with a UIS-2 20x/0.45/∞/0−2/FN22 lens (Olympus Ltd., Southend on sea, U.K.) and a Canon EOS 500D SLR digital camera were used to obtain all images. Image processing was conducted using Image J, which is freely available from http://imagej.nih.gov/ij/.

GFP Freeze/Thaw Assay for GFP Conjugates

GFP conjugate and GFP stock solutions were made up to a stock concentration of 3 $\mu g.mL^{-1}$ of protein, as measured by absorbance at 280 nm. The concentration of PVA conjugated to GFP in these solutions was approximately 2 $mg.mL^{-1}$. Conjugate and GFP solutions (200 µL) were added to a 96 well plate in triplicate and the plate sealed with SealPlate film. Fluorescence was recorded at 25 °C using a BioTek Synergy HTX multimode reader using an excitation wavelength of 395 nm and an emission wavelength of 509 nm. Samples were then frozen at -

20 °C and then thawed at ambient temperature. The fluorescence recorded as above as compared to that of the unfrozen conjugate and GFP solutions.

Ice Recrystallisation Inhibition (Splat) Assay

A 10 μL sample of polymer dissolved in PBS buffer (pH 7.4) is dropped 1.40 m onto a glass microscope coverslip, which is on top of an aluminium plate cooled to −78 °C using dry ice. The droplet freezes instantly upon impact with the plate, spreading out and forming a thin wafer of ice. This wafer is then placed on a liquid nitrogen cooled cryostage held at −8 °C. The wafer is then left to anneal for 30 min at −8 °C. The number of crystals in the image is counted, using ImageJ, and the area of the field of view divided by this number of crystals to give the average crystal size per wafer, and reported as a % of area compared to PBS control.

Recombinant Expression and Purification of Green Fluorescent Protein

A pWALDO plasmid encoding for a hexahistidine-tagged GFP was kindly provided by Elizabeth Fullam (Warwick University, Coventry, UK). The plasmid was transformed into competent Escherichia coli BL21(DE3) cells (New England Biolabs). A colony was selected to inoculate 50 mL of LB-medium containing 100 $\mu g.mL^{-1}$ ampicillin and was grown overnight at 37 °C under continuous shaking of 180 rpm. The following day, 10 mL of the preculture was added to 1 L of LB-medium (supplemented with 100 $\mu g.mL^{-1}$ ampicillin) in a 2.5 L Ultra Yield™ flask and grown at 37 °C with a shaking speed of 180 rpm till on OD_{600} of 0.6 was reached. The temperature was then reduced to 16 °C and the cells incubated for another hour before adding IPTG to a final concentration of 1 mM. The overexpression of the protein was allowed to take place overnight following which the cells were centrifuged at 5000 *g* for 10 minutes at 4 °C. Pelleted cells were resuspended in PBS supplemented with Pierce protease inhibitor mini-tablets. The suspension was passed through a STANSTED ‘Pressure Cell’ FPG12800 homogenizer in order to lyse the cells. The cell lysate was centrifuged at 48,000 *g*

and the supernatant was passed through a 0.45 μm filter before being added to a 3 mL column of IMAC complete His-Tag Purification Resin (Roche) pre-equilibrated with PBS. The column was washed with 20 column volumes of 20 mM imidazole in PBS. Bound GFP was eluted using 6 mL of 300 mM Imidazole in PBS. Further purification of GFP was achieved using a HiLoad 16/600 Superdex 75 pg gel filtration column (GE Healthcare) with PBS as the running buffer. Purity was estimated using SDS-PAGE and protein concentration determined using Thermo Scientific Pierce BCA assay kit. Various volumes of the GFP containing PBS solution were aliquoted into 1.5 mL microcentrifuge tubes and snap-frozen in liquid nitrogen to store at -80 °C till required.

Synthesis of 2-(ethoxycarbonothioyl)sulfanyl propanoate (EXEP)

Into a round bottom flask was added ethyl acetate (120 mL), potassium ethyl xanthate (4.7 g, 0.02 moles) and then dropwise 2-(methyl bromopropionate) (5.0 mL, 0.03 moles) and the solution left to stir overnight at 60°C. The mixture was filtered to remove insoluble KBr and then concentrated *in vacuo*. The crude product was partitioned into DCM (100 mL), washed with water (2x100 mL) and then brine solution (1x100 mL) and the organic phase was dried using magnesium sulphate ($MgSO_4$). The solution was filtered and concentrated *in vacuo,* affording the product as a yellow oil. ^{1}H NMR (400 MHz, $CDCl_3$): δ = 1.42 (CH_3CH2O, t, 3H), 1.57 (SCH(CH_3)CO, d, 3H), 3.75 (COOCH_3, s, 3H), 4.40 (SCH(CH_3)CO, q, 1H), 4.63 ($CH_3$$CH_2$O, q, 2H). ^{13}C NMR (400 MHz, $CDCl_3$): δ = 14 (*C*H_3CHO), 18 (SCH(*C*H_3)CO), 47 (S*C*H(CH_3)CO) , 55 (COO*C*H_3), 70 (CH_3*C*H_2O), 173 (SCH(CH_3)*C*OO), 211 (O*C*(S)S). HRMS (ESI -) m/z: 231.0 [M-H], expected 231.3.

Synthesis of Poly (vinyl acetate)

As a representative example, into a 20 mL glass vial were added vinyl acetate (VA) (3 g, 34 mmol), EXEP (0.07 g, 0.34 mmol) and ACVA (0.09 g, 0.034 mmol). The vial was sealed with

a subaseal and the solution thoroughly degassed under a flow of N_2 in ice bath for 10 min. The polymerisation mixture was then heated at 70 °C in oil bath for 8 hours. After an aliquot had been taken for conversion analysis, the sample was diluted in methanol (15 mL) and precipitated into swirling petroleum ether (2 x 200 mL). Conversion (NMR): 30%, Mn(theoretical): 1900g.mol^{-1}. ^{1}H NMR($CDCl_3$): δ = 1.72-1.97 (CH_2CHOOCH$_3$, br), 1.98-2.15 (CH$_2$CHOOCH_3, br), 4.82-5.11 (CH$_2$CHOOCH$_3$, br). Mn(SEC)$CHCl_3$: 2400 g.mol^{-1} Mw/Mn(SEC): 1.33. FTIR: C=O 1729 cm^{-1}, C-O 1370 cm^{-1}.

Reduction of poly(vinyl acetate) to poly(vinyl alcohol) using hydrazine hydrate solution

To a 50 mL round bottom flask equipped with a stir bar, poly(vinyl acetate) (1.00g) was dissolved in methanol (10 mL) and left to stir until dissolved. Hydrazine hydrate solution (25 mL, 50-60%) was added and the mixture stirred at ambient conditions for 24 h. The reaction was then concentrated in vacuo to remove unreacted hydrazine hydrate and methanol, and the residue diluted with MilliQ grade water (50 mL) and the mixture dialysed using dialysis tubing (MWCO = 1000 Da). The dialysed sample was freeze dried, affording PVA as a white powder. ^{1}H NMR (400 MHz, D_2O): δ 4.00 (CH$_2$CHOH, br, 1H), 1.68−1.60 (CH_2CHOH, br, 2H) FTIR: O-H (Alcohol) 3278 cm^{-1}, C-O 1418 cm^{-1}.

Reduction of poly(vinyl acetate) to poly(vinyl alcohol) using Sodium Hydroxide

To a 50 mL round bottom flask equipped with a stir bar, poly(vinyl acetate) (1.00 g) was dissolved in methanol (10 mL) and left to stir until dissolved. Sodium hydroxide solution (1M, 20 mL) was added and the mixture stirred at ambient conditions for 24 h. The reaction was then diluted with MilliQ grade water (50 mL) and the mixture dialysed using dialysis tubing (MWCO = 1000 Da). The dialysed sample was freeze dried, affording PVA as a white powder. ^{1}H NMR (400 MHz, D2O): δ = 4.00 (CH$_2$CHOH br 1H), δ = 1.68–1.60 (CH_2CHOH br 2H) FTIR: O-H (Alcohol) 3278 cm^{-1}, C-O 1418 cm^{-1}

Additional Data

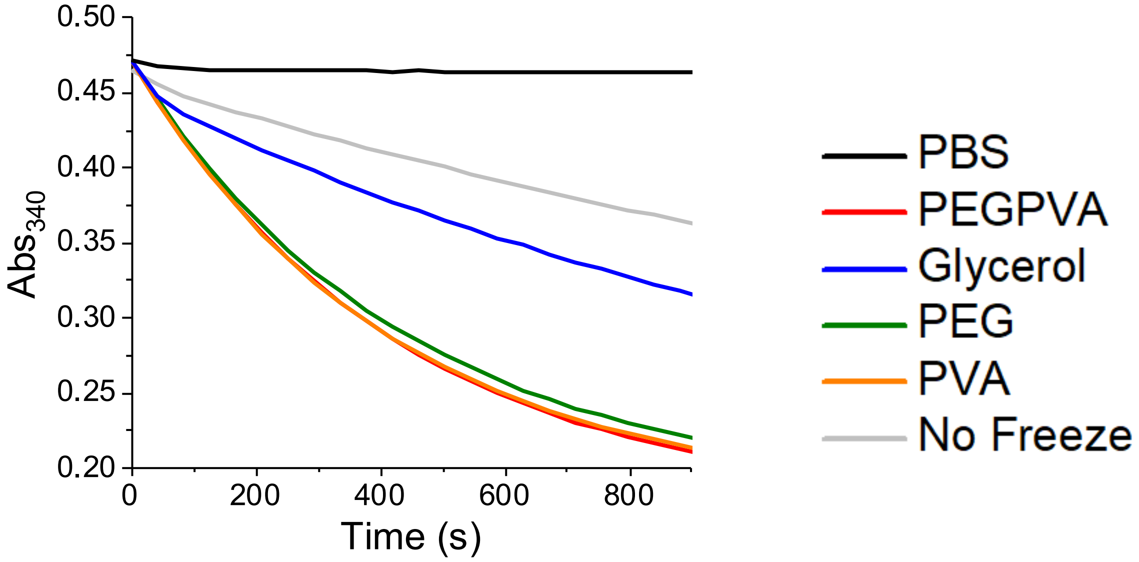

Figure S.1 Freeze-thaw recovery of LHD after 7 freeze (in Liq. N2) and thaw (25 °C) cycles. Glycerol is 25 % (v/v). PEG is 100 mg.mL^{-1} and PVA is 1 mg.mL^{-1}.

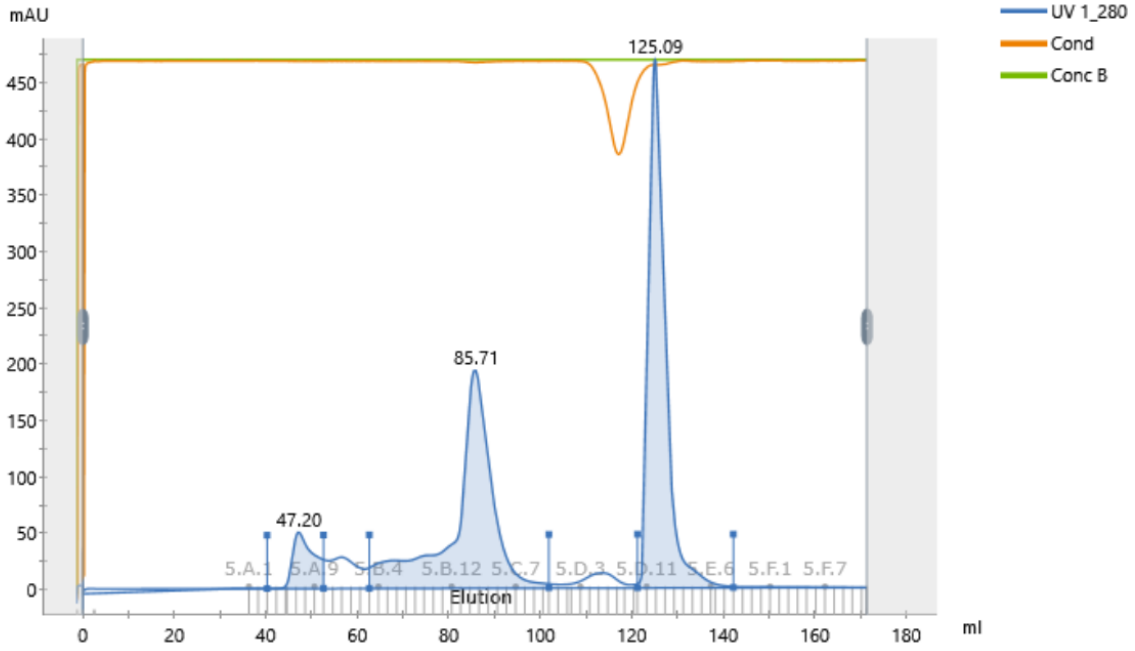

Figure S.2 FPLC trace of synthesised GFP for LDH assay.

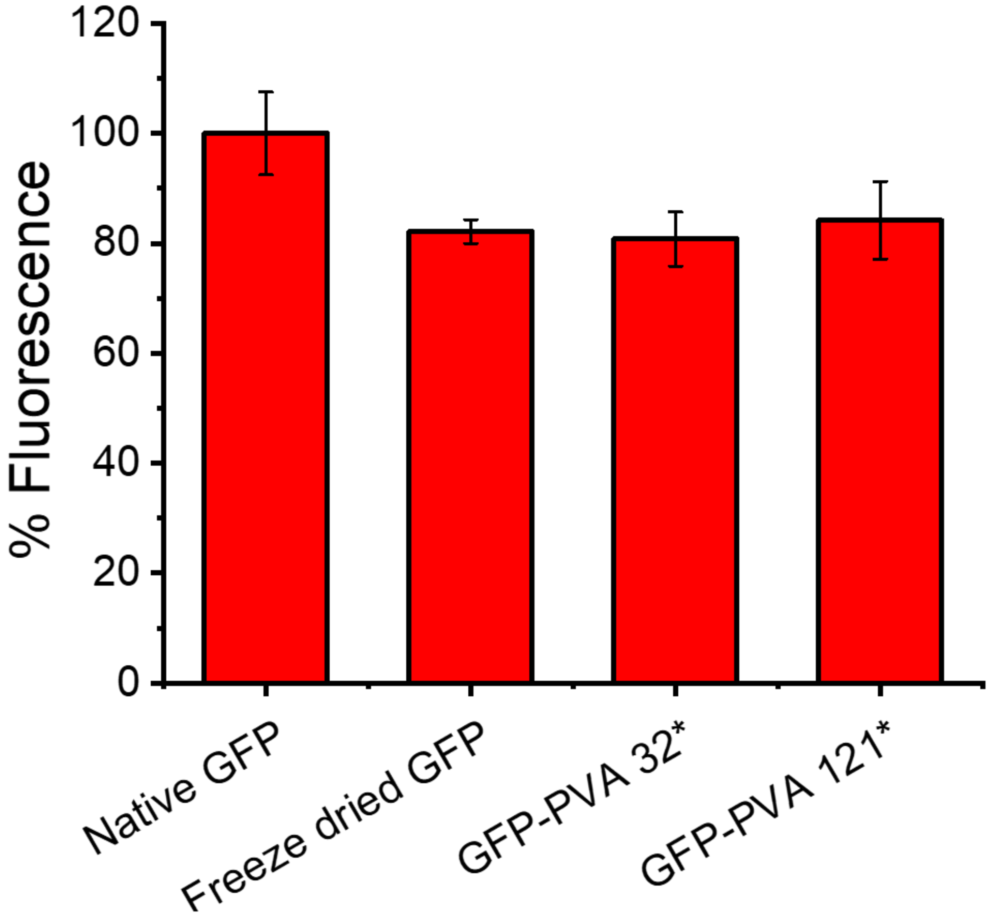

Figure S.3 Changes in GFP fluorescence after polymer conjugation. Protein concentration in all cases 1 μM in PBS buffer. Native GFP directly after synthesis and FPLC purification. Freeze dried GFP was buffer exchanged to PBS then freeze dried. GFP-PVA 32* and GFP 121* were freeze dried before any freeze/thaw cycles had been conducted.

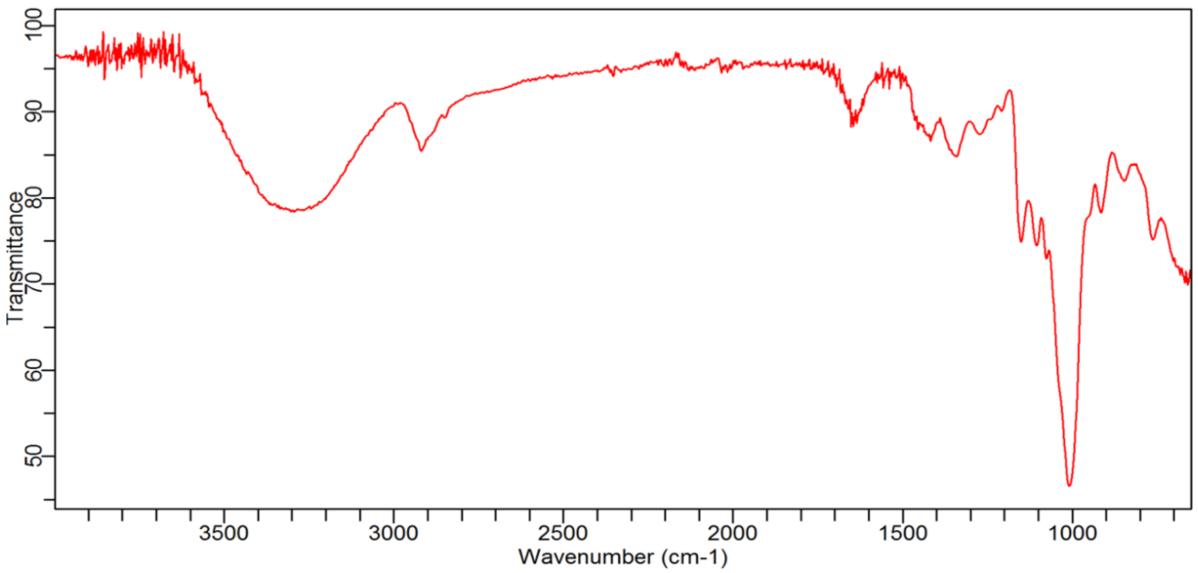

Figure S.4 FTIR of PVA 32* prepared by hydrolysis with NaOH. Characteristic carboxylate stretch at 1650-1510 cm^{-1}, in addition to O-H stretch at 3550-3200 cm^{-1} and C-H at 2950-2850 cm^{-1}.

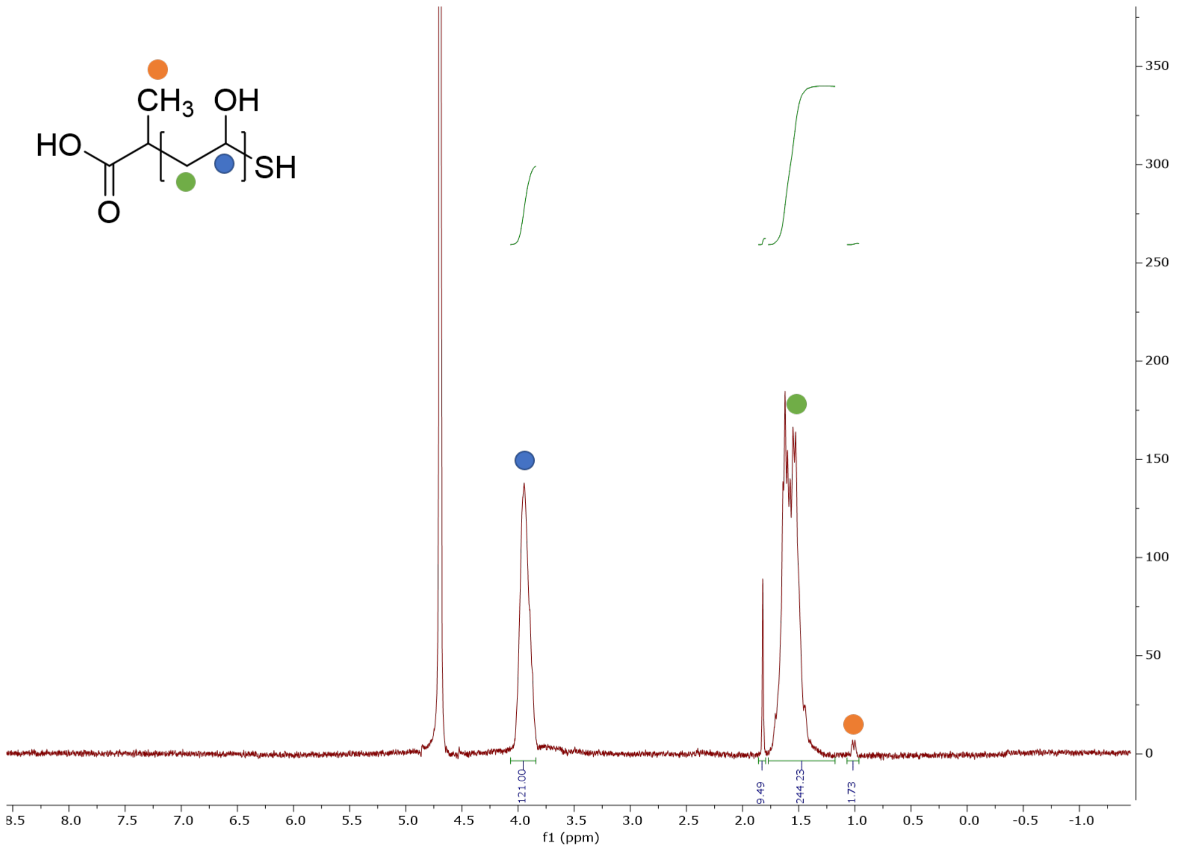

Figure S.5 ^{1}H NMR Spectrum of PVA*121 in D_2O. Residual acetate groups at 1.8 ppm show 97.5% hydrolysis has been achieved. The doublet at 1.1 ppm confirms presence of the methyl group of the PVA end group, and the lack of any defined peak at 4.8 ppm indicates that the methyl ester end group ahs been successfully hydrolyzed to a carboxylic acid.

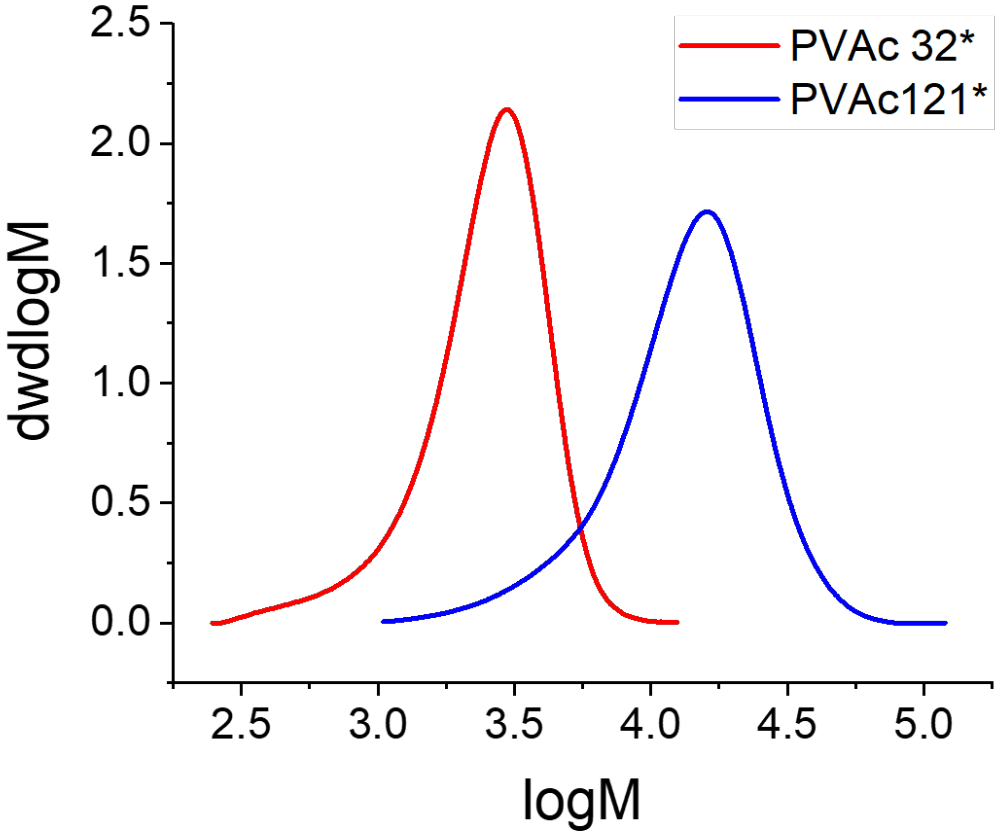

Figure S.6 Size exclusion chromatograms for PVAc 32* and PVAc 121* in THF
